# Enhanced N_2_O Production Induced by Soil Salinity at a Specific Range

**DOI:** 10.3390/ijerph17145169

**Published:** 2020-07-17

**Authors:** Yawei Li, Junzeng Xu, Boyi Liu, Haiyu Wang, Zhiming Qi, Qi Wei, Linxian Liao, Shimeng Liu

**Affiliations:** 1 State Key Laboratory of Hydrology—Water Resources and Hydraulic Engineering, Hohai University, Nanjing 210098, China; yaweizx@hhu.edu.cn; 2 College of Agricultural Science and Engineering, Hohai University, Nanjing 210098, China; boyi1027ns@163.com (B.L.); haiyu_wang@outlook.com (H.W.); weiqi8855116@163.com (Q.W.); liaolinxian@hhu.edu.cn (L.L.); threethreelsm@outlook.com (S.L.); 3 Department of Bioresource Engineering, McGill University, Sainte-Anne-de-Bellevue, QC H9X 3V9, Canada; zhiming.qi@mcgill.ca; 4 Cooperative Innovation Center for Water Safety & Hydro Science, Hohai University, Nanjing 210098, China

**Keywords:** soil salinity, N_2_O emission, soil inorganic N, nitrification

## Abstract

Nitrous oxide (N_2_O) as a by-product of soil nitrogen (N) cylces, its production may be affected by soil salinity which have been proved to have significant negative effect on soil N transformation processes. The response of N_2_O production across a range of different soil salinities is poorly documented; accordingly, we conducted a laboratory incubation experiment using an array of soils bearing six different salinity levels ranging from 0.25 to 6.17 dS m^−1^. With ammonium-rich organic fertilizer as their N source, the soils were incubated at three soil moisture (θ) levels—50%, 75% and 100% of field capacity ($\theta_{\mathrm{fc}}$)—for six weeks. Both N_2_O fluxes and concentrations of ammonium, nitrite and nitrate (NH_4_^+^-N, NO_2_^−^-N and NO_3_^−^-N) were measured throughout the incubation period. The rates of NH_4_^+^-N consumption and NO_3_^−^-N accumulation increased with increasing soil moisture and decreased with increasing soil salinity, while the accumulation of NO_2_^−^-N increased first then decreased with increasing soil salinity. N_2_O emissions were significantly promoted by greater soil moisture. As soil salinity increased from 0.25 to 6.17 dS m^−1^, N_2_O emissions from soil first increased then decreased at all three soil moisture levels, with N_2_O emissions peaking at electric conductivity (EC) values of 1.01 and 2.02 dS m^−1^. N_2_O emissions form saline soil were found significantly positively correlated to soil NO_2_^−^-N accumulation. The present results suggest that greater soil salinity inhibits both steps of nitrification, but that its inhibition of nitrite oxidation is stronger than that on ammonia oxidation, which leads to higher NO_2_^−^-N accumulation and enhanced N_2_O emissions in soil with a specific salinity range.

## 1. Introduction

With more than 8.3 × 10^6^ km^2^ of salt-affected lands globally, soil salinization is one of the most severe forms of land degradation [1]. Mostly situated in arid and semi-arid regions [2], the expanse of such lands is increasing in several regions of the world due to climate change, seawater intrusion and irresponsible irrigation and drainage management [3]. Soil salinization not only severely inhibits plant growth and production [4], but also has a profound impact on soil nutrient cycling: soil nitrogen (N) transformations are significantly altered in salt-affected soils [5,6].

Emissions of nitrous oxide (N_2_O), a by-product of soil N transformation and a potent greenhouse gas, from different ecosystems has been a hot research topic in the past decades [7,8]. Crucially, terrestrial ecosystem contributes nearly 70% of global N_2_O emissions [9]. Soil N_2_O production is primarily driven by the array of soil microorganisms associated with soil N cycles. Nitrification (including nitrifier denitrification) and heterotrophic denitrification were considered to be the main sources of soil N_2_O production and their contributions to N_2_O production were affected by soil environmental factors and N substrate types [10], which have been extensively studied in non-saline soils [11,12,13]. However, little information exists regarding N_2_O production in saline soils and only a handful of recent studies suggested that salinity affects N_2_O emissions from soils [14,15,16,17]. Reddy and Grohn reported increased N_2_O emissions at higher soil salinity level (15.2–30.6 dS m^−1^, EC of soil saturation extract) from a soil with high nitrate (NO_3_^−^-N) inputs at the beginning of the incubation [14]. Similar results were also observed by Yu et al. and Zhang et al. from soils with a low inorganic N concentration [16,17]. It is poorly documented that the response of N_2_O production to salinity in NH_4_^+^-N -rich soils, that is the exact situation for soils after chemical fertilizers. More studies are needed to deepen our understanding of the role of soil salinity on regulating soil N transformation and N_2_O emission in saline soils.

Under aerobic condition associated with low soil moisture (θ), soil N_2_O is mainly produced through nitrification [18]. Recent studies have shown that nitrifier denitrification, a branched pathway of nitrification in which ammonia (NH_3_) is oxidized to nitrite (NO_2_^−^-N) followed by the reduction of NO_2_^−^-N to nitric (NO), N_2_O or N_2_, may be the main contributor to N_2_O production when θ is near field capacity ($\theta_{\mathrm{fc}}$) [19,20]. These N transformation pathways are mainly conducted by nitrifiers and significantly correlated with the concentrations of soil inorganic N: i.e., ammonium (NH_4_^+^-N), nitrite (NO_2_^−^-N) and nitrate (NO_3_^−^-N). Soil salinity had a significant inhibitive effect on nitrifiers, with both ammonia oxidizing bacteria and nitrite oxidizing bacteria being inhibited by salt [21,22]. While Akhtar et al. reported rates of inorganic N transformation to respond differently to different soil salinity levels [23]. The response of N_2_O production to soil salinity across a wide salinity range has a paucity of reports and there is also a deficiency in knowledge regarding the relationship between N_2_O emissions and salt-affected soil N transformation. This limits our ability to predicting N_2_O emission from soils with changing salinity.

Assuming N_2_O emission could be increased by soil salinity with a specific range, and soil moisture could affect the response of N_2_O emission to soil salinities ranging from non-saline to heavily saline, a laboratory incubation experiment was conducted on soil microcosms at six salinity levels (0.25, 1.01, 2.02, 3.21, 4.92, and 6.17 dS m^−1^) and three soil moisture levels (50%, 75% and 100% of $\theta_{\mathrm{fc}}$). We monitored N_2_O fluxes and the concentrations of three forms of soil inorganic N—NH_4_^+^-N, NO_2_^−^-N and NO_3_^−^-N throughout the incubation period. The objectives of current research were: (*i*) to assess the responses of N_2_O emissions to different soil salinity levels (from non-saline to heavily saline) under different soil moisture levels; and (*ii*) to investigate the relationship between N_2_O production and inorganic N transformation processes as affected by different soil salinity levels.

## 2. Materials and Methods

### 2.1. Soil Description

Both saline and desalted soil samples were collected in June 2017 from farmland in Yancheng, China (lat. 32°44′17″ N; long. 120°52′14″ E). The saline soil was collected from a field which had been abandoned for over 6 years due to extremely high salinity. The electric conductivity (EC) of the 0–0.15 m soil profile was up to 6.4 dS m^−1^ (EC_1:5_, soil:water = 1:5). The desalted soil was from a field which was desalted using local practice (hydrological and biological methods including flooding irrigation, the leaching and drainage of the leached water, and high salt-tolerant rice cultivation) for 4 years, from 2013 onward, and the average EC_1:5_ of which was inferior to 0.3 dS m^–1^. Both soils were silty loam in texture and classified as Salic Fluvisols according to the World Reference Base for Soil Resources issued by FAO and UNESCO. The physical and chemical properties of the saline and desalted soils are listed in Table 1. The two soils were passed through a 10 mm sieve to remove large rocks and plant fragments before they were bagged and taken back to the lab. Then, the soils were air-dried to ≈ 10% gravimetric water content before being passed through a 2 mm sieve.

### 2.2. Experimental Design

Six levels of soil salinity—S1 (0.25 dS m^−1^), S2 (1.01 dS m^−1^), S3 (2.02 dS m^−1^), S4 (3.21 dS m^−1^), S5 (4.92 dS m^−1^) and S6 (6.17 dS m^−1^)—were generated by mixing two kinds of soils in different proportions (1:0, 6:1, 5:2, 1:1, 1:3, 0:1, respectively). The mixed soils were subjected to three drying-rewetting cycles (wetting with deionized water and then air-drying) in one month to achieve a more uniform distribution of salt ions and to allow the soils to reach an established equilibrium for soil microorganisms at the desired soil salinity. Previous study showed that one month was a sufficiently long for soil microorganisms to adapt to a change in soil salinity [24]. The basic properties of the mixed soils (S2, S3, S4 and S5, listed in Table 1) were measured at the beginning of the incubation. There were no significant differences in the properties (except for soil salinity and microbial biomass) for the six soil types, which implied that the interference of factors other than soil salinity were largely excluded.

The soils of all treatments were pre-incubated in darkness for 7 days at 50% $\theta_{\mathrm{fc}}$ at 25 ± 1 °C to allow the soil microorganisms to recover from the previously dry conditions [25]. Each treatment was amended with the same quantity of powdered organic fertilizer (3.0g kg^−1^ dry soil, passed through a 0.15 mm sieve) to provide C and N substrates. The organic fertilizer was made from the tail liquid of corn fermentation, which is rich in organic carbon (45.61 ± 3.43 %) and ammonium nitrogen (60.5 ± 1.3 g kg^−1^), with a total N of 7.82 ± 0.31% and pH of 5.6 ± 0.1.

Three moisture levels, 50%, 75% and 100% $\theta_{\mathrm{fc}}$, were set for each salinity level by adding different amounts of deionized water. A total of 18 treatments were used to represent six salinity levels × three moisture levels. The treated soil samples (equivalent to 150 g dry soil each) were placed in 500 mL glass bottles with sealed lids. For each treatment combination, six replicates were prepared, three for gas sampling and the remainder for soil sampling. The glass bottles remained open during the incubation except the periods of gas sampling. To offset reduction in soil moisture caused by evaporation, water was added daily to keep soil moisture at expected levels in each treatment and the added water volume daily was calculated by the weight decrement of incubation bottle. The incubation was conducted in the same environment as the pre-incubation.

### 2.3. Gas Sampling and Flux Calculation

**Figure 1:**
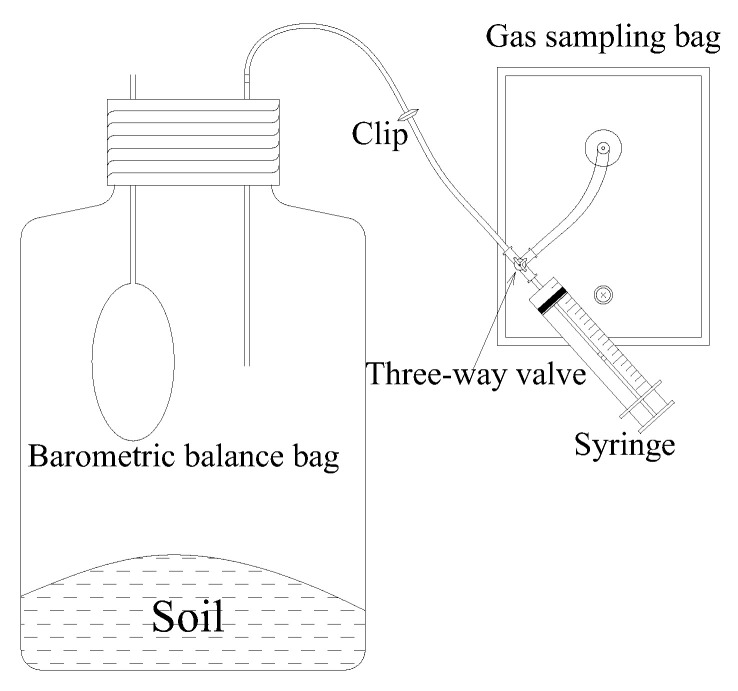
Diagram of the incubation bottle with pressure balance apparatus.

Gas samples were collected on days 0, 3, 7, 11, 16, 21, 28, 35 and 42. Prior to gas sampling, the air in the bottles was replaced with ambient air pumped at a rate of 5 L min^−1^ for 30 s, and this process was repeated three times. The bottles were sealed, and a 20 mL gas sample was immediately drawn from each bottle using a syringe through a portal connected with a three-way valve connected to the bottle lid; after 6 h another 20 mL gas was sampled. To overcome the negative pressure inside the bottle during gas sampling, a special apparatus (Figure 1) was designed to balance the pressure inside and outside the bottle.

Each gas sample was stored separately in a 30 mL gas sampling bag (Dlian Delin, Dalian, China). N_2_O concentrations in the gas samples were analyzed within 24 h after sampling using a gas chromatograph system (Agilent 7890A, Santa Clara, CA, USA). Assuming N_2_O increased linearly during 6 h for gas sampling, which was confirmed by Zhu et al. [26], the flux of N_2_O was calculated by the following equation [27]

(1) $$F=\rho \times V\times \frac{∆C}{∆t}\times \frac{273}{\left(273+T\right)\times W}$$

where, F is the flux of N_2_O (μg N_2_O-N kg^−1^ h^−1^); T is the incubation temperature (°C, T = 25 °C); V is the headspace volume inside the bottle (L, V = 0.365 L); W is the dry weight of soil in the incubation bottle (g, W = 150 g); ρ is the N_2_O density at the standard state (ρ = 1.963 kg m^−3^); ∆C is the increment in the N_2_O concentration during the sealed time period (ppmv); ∆t is the duration of the sealing time (h, ∆t = 6 h).

### 2.4. Soil Sampling and Chemical Analysis

The physicochemical properties of the six soil samples with different salinity levels were determined prior to the incubation. Soil total C and N were measured using an elemental analyzer and soil pH and EC were measured using a pH meter and an EC meter in a 1:5 soil: water mixture respectively. The concentrations of soil NH_4_^+^-N, NO_2_^−^-N and NO_3_^−^-N were measured by the colorimetric method [28], using a UV-1280 spectrophotometer (Shimadzu, Kyoto, Japan). Each soil sample (equivalent to 5 g dry soil) was shaken at 300 rpm for 1 h with 1 M KCl solution (50 mL) and filtered through filter paper (Whatman no. 42). The filtrate was used to measure NH_4_^+^-N, NO_2_^−^-N and NO_3_^−^-N concentrations. Chloroform-labile C, an indicator of soil microbial biomass, was determined by fumigation-extraction [29]. The C concentration in the filtered extracts was determined by titration with 0.033 M acidified ferrous ammonium sulphate, after adding 0.0667 M K_2_Cr_2_O_7_ and sulphuric acid. Chloroform-labile C was calculated as the difference in C concentration between fumigated and non-fumigated soil.

On the same date as the gas sampling, about 5 g soil (dry soil weight) was taken out from the remaining three bottles to measure the concentrations of soil NH_4_^+^-N, NO_2_^−^-N and NO_3_^−^-N (same method as above).

### 2.5. Statistical Analysis

Significant differences among different soil salinity levels or soil moisture levels in N_2_O emissions were analyzed using a one-way ANOVA analysis and post-hoc Fisher’s LSD test at a *p* ≤ 0.05 significant level. A two-way ANOVA was performed to test the effect of soil moisture and salinity and the interaction between them on NH_4_^+^-N consumption, NO_3_^−^-N accumulation, NO_3_^−^-N accumulation or N_2_O emissions. Statistical analysis was performed using the SPSS 19.0 software (IBM SPSS Statistics, Chicago, IL, USA).

## 3. Results

### 3.1. Soil Inorganic N dynamics

**Figure 2:**
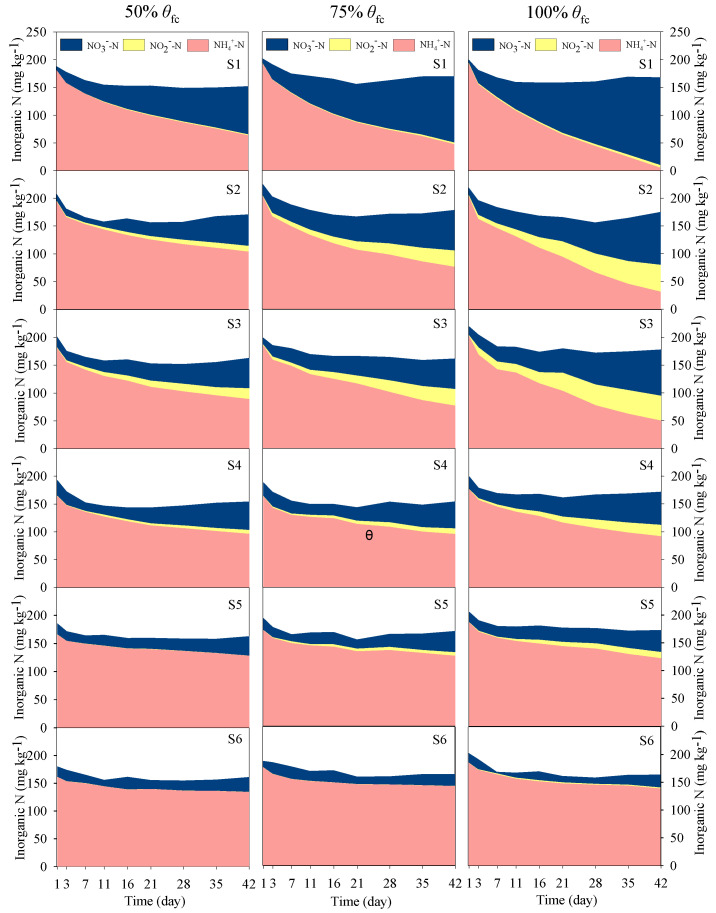
Soil NH_4_^+^-N, NO_2_^−^-N and NO_3_^−^-N concentrations in soil with different salinity levels at 50% $\theta_{\mathrm{fc}}$, 75% $\theta_{\mathrm{fc}}$ and 100% $\theta_{\mathrm{fc}}$. S1, S2, S3, S4, S5 and S6 represent six levels of soil salinity, 0.25, 1.01, 2.02, 3.21, 4.92 and 6.17 dS m^−1^, respectively. The width of color blocks represents the concentrations of NH_4_^+^-N, NO_2_^−^-N and NO_3_^−^-N respectively, each of which is the mean of three replicates.

The values of soil NH_4_^+^-N, NO_2_^−^-N and NO_3_^−^-N concentration were measured in all treatments at the onset of incubation. Soil NH_4_^+^-N were high (160–210 mg kg^−1^), but the NO_3_^−^-N were low and the NO_2_^−^-N were almost undetectable (Figure 2), which is consistent with the inorganic N composition of both the initial soil samples (Table 1) and the added organic fertilizer.

Along with the incubation, soil NH_4_^+^-N declined gradually and the NO_3_^−^-N and NO_2_^−^-N increased gradually under most treatments (Figure 2), indicating that nitrification was the main process of N transformation occurring in such incubation environment. However, the changes in soil NH_4_^+^-N, NO_2_^−^-N and NO_3_^−^-N were different among the different combined treatments. In all cases these concentrations were significantly affected by soil moisture levels. Higher soil moisture promoted the consumption of NH_4_^+^-N and enhanced the accumulation of soil NO_3_^−^-N and NO_2_^−^-N (Figure 2 and Table 2). The soil inorganic N concentrations varied differently in soils with different salinity levels. The NH_4_^+^-N consumption and NO_3_^−^-N accumulation decreased with the increasing salinity. However, The NO_2_^−^-N increased with the increasing soil salinity from S1 to S3 and decreased from S3 to S6 under all three soil moisture conditions (Figure 2).

### 3.2. Nitrous Oxide Emissions

**Figure 3:**
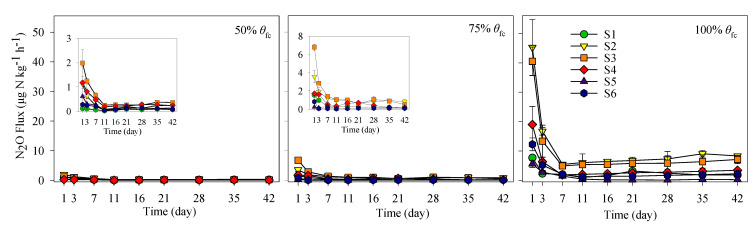
Fluxes of N_2_O emissions during the incubation period. S1, S2, S3, S4, S5 and S6 represent six levels of soil salinity (0.25, 1.01, 2.02, 3.21, 4.92 and 6.17 dS m^−1^), respectively. The inserts in 50% $\theta_{\mathrm{fc}}$ and 75% $\theta_{\mathrm{fc}}$ enlarge the ordinate to show differences more clearly. Error bars indicate standard error of mean (*n* = 3).

**Figure 4:**
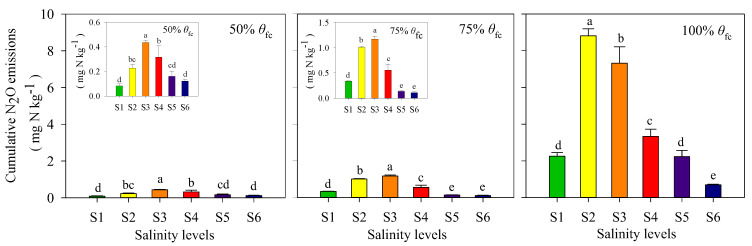
Cumulative N_2_O emissions during the incubation period. S1, S2, S3, S4, S5 and S6 represent six levels of soil salinity, 0.25, 1.01, 2.02, 3.21, 4.92 and 6.17 dS m^−1^, respectively. The inserts in 50% $\theta_{\mathrm{fc}}$ and 75% $\theta_{\mathrm{fc}}$ enlarge the ordinate to show differences more clearly. Different lowercase and uppercase letters indicate significance levels of *p* < 0.05 among salinity treatments, respectively. Error bars indicate standard error of mean (*n* = 3).

As shown in Figure 3, N_2_O fluxes followed a similar pattern over time at all three soil moisture levels. The maximum N_2_O fluxes appeared at the beginning of incubation (day 0). After a rapid drop within one week, N_2_O fluxes were relatively low and stable until the end of the incubation. Furthermore, the N_2_O fluxes increased markedly with increasing soil moisture, with differences in N_2_O fluxes among the three soil moisture levels being highly significant (*p* < 0.05). As a result, the cumulative N_2_O emissions at 100% of $\theta_{\mathrm{fc}}$ for all salinity treatments were 5.0–15.3 times greater than those at 75% $\theta_{\mathrm{fc}}$, and 4.7−37.6 times greater than those at 50% $\theta_{\mathrm{fc}}$ (Figure 4).

An interesting phenomenon is that the N_2_O emissions varied significantly across soil salinity levels. For all three soil moisture levels, the cumulative N_2_O emissions increased first then decreased with the increase of soil salinity, with peak N_2_O emissions occurring at S2 or S3. For 50% $\theta_{\mathrm{fc}}$ and 75% $\theta_{\mathrm{fc}}$, the cumulative emissions of N_2_O increased remarkably from S1 to S3 and decreased significantly from S3 to S6. The cumulative N_2_O emissions at S2 and S3 were much greater than that at other salinity levels (*p* < 0.05, Figure 4). Generally, soil salinity significantly affected N_2_O emissions (Figure 4 and Table 2), and soils with medium salinity levels (EC of 1.01 and 2.02 dS m^−1^) allowed much greater N_2_O emissions than soils with lower (0.25 dS m^−1^) or higher salinity levels (3.21–6.17 dS m^−1^). It is worth mentioning that soil microbial biomasses (measured at the beginning of the incubation) of S1, S2 and S3 were similar, but significantly lower under the S3 to S6 salinity levels (Table 1). Soil microbial biomasses from S1 to S3 were inconsistent with the N_2_O emissions (which increased significantly), although they showed a similar decreasing trend from S3 to S6.

**Figure 5:**
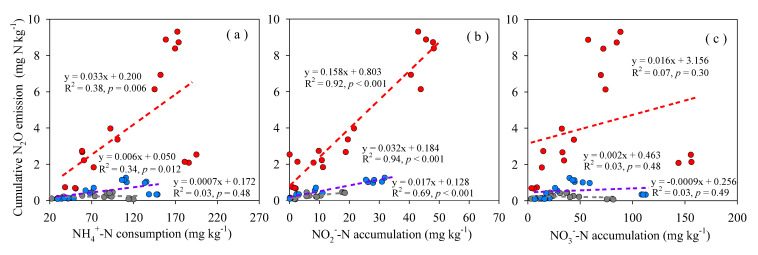
Correlation analysis of cumulative N_2_O emissions with NH_4_^+^-N consumption (**a**), NO_2_^−^-N accumulation (**b**) and NO_3_^−^-N accumulation (**c**). Gray, blue and red dots were from 50% $\theta_{\mathrm{fc}}$ 75% $\theta_{\mathrm{fc}}$ and 100% $\theta_{\mathrm{fc}}$ respectively.

**Figure 6:**
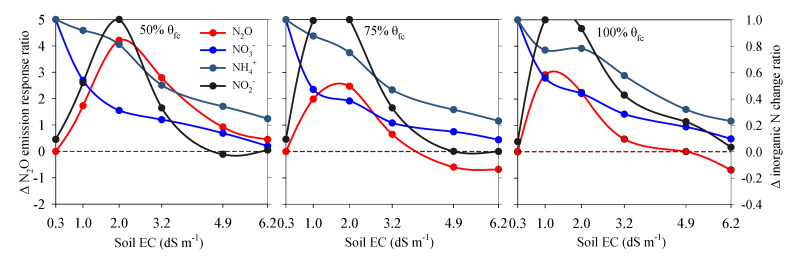
The response of cumulative N_2_O emissions, NH_4_^+^-N consumption, NO_2_^−^-N accumulation and NO_3_^−^-N accumulation to soil salinity at three soil moisture levels. N_2_O emission response ratio to soil salinity was calculated as the relative increment in N_2_O emission compared with the soil at S1 salinity level. Δ inorganic N change ratio including ΔNH_4_^+^-N consumption ratio, ΔNO_2_^−^-N accumulation ratio and ΔNO_3_^−^-N accumulation ratio, were calculated as the relative decrement or increment of respective concentration under each salinity level compared with the maximum value of that among six salinity levels.

Cumulative N_2_O emissions were found positively correlated to NO_2_^−^-N accumulation under all three soil moisture levels (Figure 5b, with R^2^ = 0.69, 0.94 and 0.92 respectively). It can be explained from the similar response of NO_2_^−^-N accumulation ratio and N_2_O emissions to increasing salinity (Figure 6). However, correlations between N_2_O emissions and NH_4_^+^-N consumption or NO_3_^−^-N accumulation was relatively weak under all three soil moisture levels (Figure 5a,c), which could be also inferred from their differences in response to increasing salinity (Figure 6).

## 4. Discussion

Under the incubation conditions employed relatively aerobic conditions (soil moisture was at or below the 100% $\theta_{\mathrm{fc}}$) prevailed and soils were rich in NH_4_^+^-N and low in NO_3_^−^-N. Such soil conditions had been shown to be suitable for nitrifiers [18,30,31,32]. Huang et al. reported that soil moisture of 100% $\theta_{\mathrm{fc}}$ was too low to trigger denitrification, in soil with moisture below 100% $\theta_{\mathrm{fc}}$ nitrification dominated soil N transformations and N_2_O was the major gaseous product [31]. Zhu-Barker et al. reported a significantly higher N_2_O emission after a NH_4_^+^-based fertilizer application comparted to a NO_3_^−^-based fertilizer addition of the same magnitude (98 kg N ha^−1^) in a field experiment with a soil moisture range of 40–80% WFPS, which indicated nitrification was likely the main source of N_2_O production from soil with NH_4_^+^-based fertilizer application [33]. Generally, with increase in soil moisture, as an alternative indicator of soil O_2_ availability [34], decreased O_2_ availability will inhibit the nitrification in soils [31]. In current research, the consumption of NH_4_^+^-N, NO_3_^−^-N accumulation and N_2_O emission at 100% $\theta_{\mathrm{fc}}$ were the highest (Figure 2 and Figure 4). It implied that soil oxygen is not a limited factor to nitrification and N_2_O emission in such soil moisture condition. Furthermore, soil NH_4_^+^-N and NO_3_^−^-N dynamics (Figure 2 and Figure 6) showed that nitrification rate increased significantly with increasing soil moisture from 50% $\theta_{\mathrm{fc}}$ to 100% $\theta_{\mathrm{fc}}$, simultaneously the cumulative emissions of N_2_O at 100% $\theta_{\mathrm{fc}}$ were dozens of times higher than that at 50% $\theta_{\mathrm{fc}}$ (Figure 4 and Table 2). As reported by many studies, ammonia oxidation and nitrifier denitrification driven by nitrifiers are the main sources of soil N_2_O when soil moisture is below 100% $\theta_{\mathrm{fc}}$ [19,20,32,35,36]. Considering that the increases in consumption of NH_4_^+^-N with soil moisture (Figure 2), it can be inferred that nitrification including nitrifier denitrification was the main sources of soil N_2_O in current soil condition.

Soil salinity is an important variable in affecting soil nitrification process, thereby may affecting N_2_O emissions from nitrification. In current study, it was found that N_2_O emission was significantly affected by soil salinity (Table 2), and the responses of N_2_O emissions to soil salinity were found varied among different salinity ranges (Figure 6). The cumulative N_2_O emissions increased at first and then decreased with increase in soil EC from 0.25 dS m^−1^ to 6.17 dS m^−1^ at all three soil moisture levels (Figure 4). It indicated that soil salinity within a certain range will promote N_2_O production, and this range was found about 1.01–2.02 dS m^−1^ in current soil. The enhanced N_2_O emissions was supported by some studies on salt-affected soils [14,16,17,37,38], most of which ascribed it to the inhibition on N_2_O reductase by salt. Soil conditions with relatively anaerobic conditions or high NO_3_^−^-N availability were more suitable for denitrifiers who contributed almost all of total N_2_O production [14,38]. However, the inhibitory effect of salt on N_2_O reductase may not explain our results which likely indicated that there are some other reasons for the enhanced N_2_O emissions from soils at a specific range of soil salinity. At the beginning of the incubation, soil microbial biomasses of S1, S2 and S3 were similar (Table 1), which means that the increase in N_2_O emission may not be attributed to soil total microbial biomasses, but specific microbial abundance or enzyme activity affected by soil salinity [31]. Soil microbial biomass showed a similar reduction as N_2_O emission from S3 to S6 (Figure 4 and Table 1), which implied reduction of total microbial population may limit soil N transformation and N_2_O production.

The different degree of response of NH_4_^+^-N consumption, NO_2_^−^-N accumulation and NO_3_^−^-N accumulation to soil salinity likely indicated that ammonia oxidation and nitrite oxidation respond to soil salinity in different magnitudes (Table 2, Figure 2 and Figure 6), which means that they are different in sensitivity to soil salinity. This may be an important reason for the promoted N_2_O emissions from soils with EC of 1.01–2.02 dS m^−1^. In non-saline soils (such as S1, EC = 0.25 dS m^−1^), along with the consumption of NH_4_^+^-N, NO_3_^−^-N was accumulated, NO_2_^−^-N was not. When it comes to soils S2 and S3 (EC = 1.02 dS m^−1^ and 2.02 dS m^−1^), NO_2_^−^-N was accumulated in a much higher degree than under S1 (Figure 2). This result implied that nitrite oxidation was inhibited to a larger degree than ammonia oxidation by soil salinity. Similar results were observed by Akhtar et al. who found NO_2_^−^-N was accumulated in soil mixed with urea and ammonium sulfate at medium or high salinity [23]. Some studies focusing on microbial nitrification process found that ammonia-oxidizing bacteria (AOB) was more tolerant than nitrite- oxidizing bacteria (NOB) to salinity, which often resulted in NO_2_^−^-N accumulation [22,39,40]. In fact, in wastewater treatment system, salt addition could eliminate NOB and maintain AOB, thereas establish a short-cut nitrification system, which strongly demonstrates that NOB was more sensitive to salinity [41,42]. In the process of nitrification, NO_2_^−^-N is an immediate substrate for N_2_O produced via nitrifier denitrification [11,31,43]. Some studied reported that soil NO_2_^−^-N was highly correlated to N_2_O production [31,44,45,46], which were consistent with our result (Figure 5b). The large gap in the ratios between NH_4_^+^-N consumption and NO_3_^−^-N accumulation led to the accumulation of soil NO_2_^−^-N in soils at a salinity range of 1.01-2.02 dS m^−1^ (Figure 6). Meanwhile, the accumulation of soil NO_2_^−^-N may have enhanced nitrifier denitrification, thereby increased the N_2_O production and emissions in S2 and S3.

This incubation experiment enabled us to investigate the effects of soil salinity on inorganic N transformations and N_2_O emissions under different soil moisture conditions. In open field conditions, N_2_O emissions may be further affected by various environmental factors (such as soil water evaporation, soil temperature, soil redox potential and other factors). Nevertheless, the current study suggested that soil salinity within a certain range has a significant effect on nitrification process and the potential risk of enhanced N_2_O emissions. For agriculture based on salt-affected soils, some potential practices such as nitrification inhibitor should be examined to reduce salinity-induced N_2_O emissions. Moreover, future long-term field study will help to evaluate N_2_O emission risk and understand the potential function of soil salinity management to mitigating N_2_O emissions from salt-affected soils.

## 5. Conclusions

Nitrous oxide emissions were found significantly increased with an increasing soil salinity within a certain range of 1.01–2.02 dS m^−1^, although it increased in different magnitudes under three soil moisture levels. Along with it, increasing soil salinity decreased the rates of NH_4_^+^-N consumption and NO_3_^−^-N accumulation, also caused the accumulation of NO_2_^−^-N at a salinity range of 1.01–2.02 dS m^−1^. Different sensitivities of ammonia oxidation and nitrite oxidation to soil salinity accounted for the accumulation of NO_2_^−^-N, as well as the enhanced N_2_O emissions that may be the direct result of nitrifier denitrification. Our observations confirm that soil salinity is a key factor for soil N transformations and N_2_O emissions, and more studies are needed to evaluate and model the effect of salinity to soil N cycles and reactive N emissions including N_2_O in saline agriculture.

## Figures and Tables

**Table 1:** Properties of desalted soil (S1), saline soil (S6) and mixed soils (S2, S3, S4 and S5) at the beginning of the incubation.

Soil Parameters	S1 (Desalted Soil)	S2	S3	S4	S5	S6 (Saline Soil)
Sand (%)	23.12 ± 0.06	23.02 ± 0.07	22.97±0.08	22.87 ± 0.06	22.84 ± 0.19	22.67 ± 0.11
Silt (%)	62.32 ± 0.28	63.24 ± 0.79	63.01 ± 0.57	64.94 ± 0.65	64.98 ± 0.95	65.25 ± 0.73
Clay (%)	14.57 ± 0.23	13.74 ± 0.72	14.02 ± 0.52	12.19 ± 0.61	12.18 ± 0.12	12.08 ± 0.65
Bulk density (g cm^−3^)	1.34 ± 0.09					1.35 ± 0.12
Initial gravimetric moisture (%)	19.23 ± 1.06					16.18 ± 1.51
Field capacity (gravimetric, %)	24.08 ± 0.25					24.05 ± 0.18
EC1:5 (dS m^−1^)	0.25 ± 0.03	1.01 ± 0.08	2.02 ± 0.13	3.21 ± 0.16	4.92 ± 0.26	6.17 ± 0.49
pH	7.66 ± 0.03	7.71 ± 0.05	7.69 ± 0.05	7.75 ± 0.02	7.78 ± 0.06	7.83 ± 0.11
Total C (g kg^−1^)	3.54 ± 0.26	3.52 ± 0.28	3.01 ± 0.30	2.88 ± 0.17	2.74 ± 0.11	2.41 ± 0.23
Total N (mg kg^−1^)	350.3 ± 23.4	303.4 ± 18.8	310.7 ± 25.1	254.3 ± 16.3	212.4 ± 20.3	213.2 ± 15.4
NH_4_^+^–N (mg kg^−1^)	42.52 ± 3.51	40.12 ± 2.84	38.53 ± 3.02	30.23 ± 1.09	28.41 ± 3.01	25.38 ± 1.12
NO_3_^−^–N (mg kg^−1^)	3.46 ± 0.09	3.86 ± 0.12	3.67 ± 0.21	3.82 ± 0.20	3.94 ± 0.19	4.07 ± 0.27
NO_2_^−^–N (mg kg^−1^)	0.27 ± 0.02	0.35 ± 0.02	0.35 ± 0.03	0.53 ± 0.02	0.59 ± 0.05	0.75 ± 0.05
Soil microbial biomass (mg C kg^−1^)	153.2 ± 8.4	149.3 ± 5.3	150.6 ± 5.6	138.3 ± 6.6	113.4 ± 4.9	98.5 ± 6.3

**Table 2:** Two-way ANOVAs for NH_4_^+^-N consumption, NO_2_^−^-N accumulation, NO_3_^−^-N accumulation and N_2_O emissions. Different lowercase letters indicate significance levels of *p* < 0.05 among salinity treatments, respectively.

Factor	NH_4_^+^-N Consumption		NO_2_^−^-N Accumulation		NO_3_^−^-N Accumulation		N_2_O Emissions	
	F	*p-Value*	F	*p-Value*	F	*p*-*Value*	F	*p*-*Value*
Moisture	161.0	<0.001	347.3	<0.001	102.0	<0.001	511.2	<0.001
Salinity	318.5	<0.001	523.2	<0.001	403.0	<0.001	84.8	<0.001
Moisture × Salinity	10.3	<0.001	49.4	<0.001	17.4	<0.001	54.0	<0.001
